# Utilizing the common sense model to explore African Americans’ perception of type 2 diabetes: A qualitative study

**DOI:** 10.1371/journal.pone.0207692

**Published:** 2018-11-21

**Authors:** Olayinka O. Shiyanbola, Earlise C. Ward, Carolyn M. Brown

**Affiliations:** 1 Division of Social and Administrative Sciences, University of Wisconsin-Madison, Madison, WI, United States of America; 2 Department of Nursing, University of Wisconsin-Madison, Madison, WI, United States of America; 3 Division of Health Outcomes and Pharmacy Practice, University of Texas-Austin, Austin, TX, United States of America; Chinese Academy of Medical Sciences and Peking Union Medical College, CHINA

## Abstract

Illness perceptions, which are likely influenced by patients’ cultural contexts, are associated with disease self-management and adherence. African American patients perceptions of type 2 diabetes is not well understood and no known studies has used a comprehensive evidence-based theoretical framework to explore what AAs with type 2 diabetes know, believe, and think about type 2 diabetes. Understanding perceptions of an illness shared by a group of people will be useful in developing culturally-appropriate interventions targeted to the needs of the community. The purpose of this study is to explore African Americans’ perceptions of type 2 diabetes based on the common sense model of illness and self-regulation. Using a phenomenology qualitative approach and purposive sampling, 40 African American men and women, age 45–60 years old with diagnosed type 2 diabetes at least one year prior, and who took at least one prescription diabetes medication, participated in six semi-structured 90-minute focus groups conducted in a private space. Qualitative content analysis was conducted to explore African Americans beliefs about type 2 diabetes. Participants expressed that historical issues, e.g., slavery, healthcare providers, the government, and God influenced how they developed diabetes. Participants reported a loss of autonomy, a change of their identity as an employee, a social individual and sexual person, as well as anger and frustration due to having diabetes. Diabetes made the African American family bonding experience of eating difficult, and the disease diminished their cultural experiences. Concerns about diabetes ranged from fear of death and amputations to the inability to prevent the disease among their children/grandchildren. Participants perceived that medications, faith in God, and positive thinking about survival helped control diabetes. **Conclusions**: Improved diabetes self-management and medication adherence may depend on the meaning African Americans attach to diabetes, available psychosocial support for managing diabetes, and African Americans experience with diabetes.

## Introduction

Over the past several decades, the prevalence of diabetes among adults has increased, with about one-third of the United States population affected by diabetes.[1] Diabetes disproportionally affects minority individuals, especially African Americans (AAs). The risk of diabetes is 77% higher among AAs than among non-Hispanic white Americans. [2] AAs with diabetes experience greater complications and disability compared to whites with diabetes. For example, AAs with diabetes are almost 50% more likely to develop diabetic retinopathy, 1.5 times more likely to be hospitalized, and 2.7 times more likely to die from diabetes than non-Hispanic whites.[1, 2] In addition, AAs are five times more likely to develop diabetes-related kidney disease and suffer leg amputations compared to whites [3–5]

Medication nonadherence (not taking medicines as prescribed by a healthcare provider) [6] is a complex behavior that is prevalent among AAs with type 2 diabetes (12% lower adherence compared to white non-Hispanics).[7] To help patients improve adherence behavior, health care providers need to be aware of how different cultural contexts influence patients’ knowledge and beliefs about diabetes. [8, 9] Studies have shown that illness perceptions influence whether patients take their medicines or not, and hence should be taken into account in designing adherence interventions.[10–12] However, AA patients’ perception of type 2 diabetes is not well understood, and to date, no known studies have used a comprehensive evidence-based theoretical framework to explore what AAs with type 2 diabetes know, believe, and think about type 2 diabetes. Moodley et al., 2000, have called for qualitative studies that use comprehensive theories, since these type of studies are limited among AAs. [13] This qualitative study uses Leventhal’s common sense model (CSM) [14, 15] as the guiding framework to explore AAs’ perception of diabetes.

### Theoretical framework

#### The common sense model

The CSM provides a framework to understand how patient perceptions of illness influence behavior. Patients respond to a specific illness experience based on their beliefs and knowledge. [16–18] These beliefs may be constructed by their interaction with the sociocultural environment, past experiences, and their conscious and implicit knowledge of what the disease means. According to the CSM, patients create mental representations of their illness to “make sense” of their experience with the disease and to manage the problem. [19] These illness representations are personal beliefs and perceptions that contribute to the individual’s action towards engaging in disease self-management behaviors such as medication adherence.

Patient illness perceptions are formed based on knowledge obtained from their sociocultural environment (e.g., friends and family), from influential sources such as health care providers, and from individual personal experience of the illness (e.g., symptoms or anxiety associated with the disease).[20] The CSM illness perceptions are characterized by five domains including *identity*, *timeline*, *cause*, *cure/control*, *and consequences*.[21, 22] Identity refers to the belief a person holds regarding the label of their symptoms and the illness they are experiencing. Timeline refers to beliefs about how long the illness will last and whether the illness is an acute or chronic illness. Cause reflects the person’s beliefs about the cause/etiology of the illness. Cure/control of an illness refers to beliefs about whether an illness can be cured or controlled based on personal efforts or the use of treatment. [20] Consequences reflect individual beliefs about possible effects the illness will have on the patient’s life. Emotional representations such as fear, anger, or anxiety are also integral to illness perceptions and develop simultaneously with other components of the CSM.

Prior studies have examined how different patient racial groups with diabetes perceive their illness, including Mexican Americans [23], and overweight AAs and European Americans. [24] Only one study was identified that explored the illness narratives of AA men with type 2 diabetes.[25] However, this study only focused on symptom symbols, patient and family explanatory models, culturally-marked disorders, and patient personal and interpersonal significance of diabetes. Skelly et al., 2006 examined the beliefs about diabetes among poor AAs living in a rural area who were at high risk for diabetes. These AAs were not currently diagnosed with diabetes.[8] To our knowledge, this is the first study to extensively explore the illness perceptions of AA men and women diagnosed with type 2 diabetes. Skelly et al., 2006, suggested that involvement in the medical system might change what individuals know and believe about diabetes. Hence, our current study examines how patients currently diagnosed with type 2 diabetes view their illness, as their illness perceptions are likely to be associated with their medication-taking behaviors and diabetes self-management efforts.

Illness perceptions predict diabetes self-care behavior including blood glucose monitoring, medication adherence, and glycemic control. [26–28] Understanding how AAs view their illness is significant in understanding patient decisions to accept and use treatment, including medications. [29] Some studies [13, 30] have noted that black communities use a different conceptual model to represent their illnesses. Understanding perceptions of an illness shared by a group of people will be useful in developing culturally-appropriate interventions targeted to the needs of the community. Healthcare providers can provide tailored interventions that address negative beliefs about diabetes influencing medication adherence. In addition, eliciting AA patients’ illness perceptions could improve provider-patient communication by enhancing understanding between both parties, which may in turn reduce frustration, and poor health care outcomes. [29] To this end, the objective of this study was to utilize the rigorous theoretical framework of the common sense model to explore AAs beliefs about type 2 diabetes.

## Materials and methods

### Research design

AA perceptions of the label/identity, timeline, cause, cure/control, and consequences of type 2 diabetes (CSM domains) were explored using an exploratory descriptive qualitative approach [31, 32] and focus groups. Qualitative methods provide rich and detailed information about how individuals experience and understand life events.[33] Such focus groups allow participants to elaborate on their illness beliefs and allow expansion on group responses [34], in addition to eliciting emotional processes related to having diabetes.

### Sample

A purposive sample of English-speaking AA men and women 45–60 years old with type 2 diabetes were recruited. The study objectives focused on these individuals because type 2 diabetes is responsible for 95% of the diabetes in AAs.[35, 36] Adults ≥ 45 years old are more frequently diagnosed with type 2 diabetes. [37, 38] The inclusion criteria were self-identified English-speaking African American/Blacks, self-reported type 2 diabetes diagnosed by a health provider at least 1 year prior, and self-reported use of oral diabetes medications. Patients with this length of diabetes diagnosis would have enough experience to have developed illness beliefs. [39–41] Also, among adults taking diabetes medications, 58% only take oral medications, while 12% only take insulin.[38] Since medication adherence and treatment beliefs differ based on the type of diabetes medication used, [42] the sample included individuals only taking oral medications.

### Measures

#### Demographic and clinical characteristics questionnaire

Demographic information including age, gender, highest level of education completed, annual household income, and health insurance status was collected. Clinical characteristics collected included self-reported health status, number of daily prescription medicines, number of chronic illnesses, and number of years of diabetes diagnosis.

#### Focus groups and interview guide

Guided by the common sense model, the focus group interview guide was comprised of open-ended questions based on the illness perception domains within the CSM (See Appendix 1).

### Recruitment

The recruitment of study participants occurred in different community sites in two cities in a US Midwestern State. Community locations included federally qualified community health centers, senior centers, apartment complexes, and a church. The investigators placed the flyers in these locations, and an investigator worked with key informants in community establishments to recruit patients. In addition, by working with AA community advisory boards and multiple community networks, the study information was shared by word of mouth. The Health Sciences Institutional Review Board of the primary investigators’ university approved the study.

### Data collection

Six semi-structured 90-minute focus groups were conducted at two locations within the state to include diverse perspectives. The first location was a city of about 200,000 people (all three focus groups in a community center) and the second was a city of about 2 million people (three focus groups: one in a church, one in an apartment complex, and one in a senior center). At the beginning of the focus groups, the authors obtained written informed consent and participants completed a brief three-minute questionnaire that examined their sociodemographic and clinical characteristics. For each discussion group, participants talked about their beliefs about diabetes. The principal investigator, with input from the study co-investigators, developed the discussion questions.

The focus groups were audio recorded and facilitated by the principal investigator (AA PhD trained female individual with extensive experience conducting focus groups) and a trained AA project assistant (AA bachelor degree graduate with three years of community-based field work) who took notes. Consistent with focus group methodology, the moderator used qualitative research skills to redirect discussions back to the main topic as necessary, prevent stronger participants from exerting control over other participants’ verbalization, and engage quiet participants.[43] All data collection was iterative, allowing for continual refinement of the data. Participants received a $50 cash incentive upon completion of a focus group.

### Data analysis

All focus groups were audio recorded and transcribed verbatim by a certified professional transcriptionist. Six transcripts made up the data for analysis. Two researchers, both of whom were skilled in qualitative research, analyzed the data. To ensure accuracy of the transcripts, a research team member verified the transcripts against the audio recordings. Using the transcripts, a qualitative content analysis was conducted. NVivo 10 (QSR International-Melbourne) was used to organize and categorize themes. First, a deductive coding framework based on the illness beliefs domains within the CSM was used to classify participants’ responses according to ‘a priori’ coding categories. Second, an inductive approach using open coding (involves reading the transcripts repeatedly, developing a coding frame for the emerging categories and conceptualizing it into themes) [44], allowed identification of newer items prompted during the discussion. A comparison of themes across individual participant responses and groups helped explore the similarities, differences, and interconnections across the codes and participants. Documentation of emerging relationships between themes was done. All analysis occurred until data saturation, when no new dimensions emerged within the data.[31, 45, 46] The members of the research team coded all transcripts independently. Subsequently, similarities and divergences were discussed. Agreement was reached on all codes before the interpretation of results. After completing the analysis, to check for accurateness and resonance with participant experiences, the results section of the manuscript was given to four focus group participants to conduct member checking.[47] Member checking involves returning the analyzed data to a participant to verify the trustworthiness of the qualitative results and to enhance the rigor of the data analysis process.[48] These individuals were selected because of their indicated interest in future and related research opportunities. Participants agreed with the results, reported their experiences were captured in the results, and did not recommend any changes or additions.

## Results

### Sample information

Forty AA men and women participated in the study. The majority of participants were female (n = 25, 61.4%) with a mean age of 53.3 (±4.9) years. Most participants had a high school degree (n = 11, 28.2%) or some college education (n = 13, 33.3%), and self-reported that their health status was fair (n = 17, 43.6%). The mean total number of daily prescription medications used was 7.2 (±6.2) and the mean number of chronic illness experienced by participants was 2.9 (±1.8). Participants had a mean duration of diabetes diagnosis of 9.6 (±7.2) years.

### Perceptions of diabetes

Participant perceptions of type 2 diabetes were based on five CSM domains outlined in the sub-headings below. Other perceptions related to emotional representations of diabetes including patient concerns about the disease.

- Label of the symptoms experienced (before and after diabetes diagnosis).
- Perception of the timeline of diabetes
- Perceived cause of diabetes
- Perception of treatment and personal control of diabetes
- Perceptions of the consequences of diabetes

#### CSM domain: Participants’ label of the symptoms experienced before diabetes diagnosis and after diabetes diagnosis

Participants identified a variety of symptoms that they experienced before diabetes diagnosis and after the diagnosis of diabetes (Table 1).

**Table 1:** African Americans perception of symptomatology related to type 2 diabetes.

Timeline of symptomatology	Symptom (s)	Sample quotes
**Before diabetes diagnosis**	Feeling hot in cold weather Dry mouth Excessive thirst	**0020**: when I was diagnosed as a diabetic, I didn't know. I was at work and I took sick, and I was cold. Then I was hot. It was in the wintertime. Even though the heat was on, you know, and I was drinking water constantly…my mouth was dry, and I was cold, and I was hot. So I went to the hospital and he told me that I was a diabetic
	Fatigue and tiredness	**0001**: ..Very thirsty… but my experience was that I started losing sleep..because I was running to the bathroom every 30 minutes, so I was getting like physically exhausted. And I still didn't know that it was diabetes.
	Change of skin color Hair thinning Boils	**0018**: my skin color changed, got two shades lighter, my hair thinned out, came out. I was always tired and lethargic and lazy, and, you know, then I had the boils in my vagina from my sugar being so high. I didn't know what was going on. So, finally, my mom took me to the clinic, and the lady's like, does diabetes run in your family? I'm like, yeah, on my dad's side.
	Excessive/Frequent urination	**0006**: And sometimes . . . I would be scared to go somewhere if I didn't have something to drink right away. But going to the bathroom was like every ten minutes, …Every half hour I was like I stopped the truck, go use the bathroom. And what I was noticing is like every time I go on a call, I would have to use the bathroom. I didn't put the two together.
	Vision changes	**WOMAN:** Well, the reason I knew because I couldn't see.
	Decreased mobility	**MAN 2:** I was walking through the kitchen and banged up against the walls. I couldn't walk. I said, something wrong. I have to go to the doc.
	Change in food taste	**DBPID_0041:** A lot of food was tasting funny. It wasn't tasting like it normally tastes..
	Sticky urine	**DBPID_0038:** So my girlfriend, she told me, she said, you know what? She said you might be a diabetic. I said, why you say that? She said because you went to the bathroom, and you didn't wipe the toilet off this time, she said, and it was kind of sticky.
**After diabetes diagnosis**	Body odors	**0011**:.., that's another telltale about your diabetes. But you got to learn how to smell your body. Your body gives off odors if it's sick.
	Polyuria and Weight loss	**0005**: When I first got diagnosed, I was like (name)… I started to lose some weight. My first symptom was a polyuria. That's going to the bathroom. Then I dropped 23 pounds. I wasn't trying to lose it, so my doctor that found it..And I keep going, constantly going to the bathroom. Every time I do it's like, oh, I got to go. And since, ..polyuria..do this and then you run into the bathroom.
	Sugar cravings	**0006:** when you have diabetes…you crave sugar. You try to stay away from it, but you got this craving, and you want to eat some sugar, and you wanted bad…in the middle of the night, I was just wanting to get up and go to the store and get something sweet. I just need something sweet.

#### CSM domain: Perception of the timeline of diabetes

Participants believed that diabetes would last for a while and there was no cure for the disease. On the other hand, some participants reported that diabetes would go away with exercise and weight loss.

*DBPID_0038: …it don't really bother me no more, because there's nothing I can do with it to make it go away. The only one thing I can do is to take my medicine faithfully*.

*DBPID_0025: I know some people that were a diabetic, they got a good, healthy, balanced diet and went to the gym, and they're no longer on medicine or anything anymore*. *I'm believing that it'll go away for me one day*

#### CSM domain: Perceived cause of diabetes

In general, participants were aware of the multicausality of diabetes. Yet, while some participants believed diabetes was hereditary and caused by eating habits, others were confused about the cause(s) of diabetes. For example, participants stated:

*DBPID_0029: My brother and sister ain't got it*. *And, man, how did I end up with it, and they didn't?*

*DBPID_0014: … that's something that the doctor should have been explaining to you why you can get diabetes, and you sit there in the same household, eating the same food… my husband, he eat candy like it's going out of style, you know, and eat everything in sight, and he don't have diabetes. I eat less than him, and I eat more fruits daily, vegetables, a little bit more of it … than he do, and … he just eats crazy stuff. And ain't nothing wrong with that man, like I say, that's not explained to us*.

Perceived cause of diabetes was classified into general reasons, biology and genetics, psychological, environment, medications, and sociocultural specific factors related to AAs. The sociocultural reasons AAs perceived for diabetes included the eating of AA cultural food and historical circumstances of AAs that led to unhealthy diets. Participants suggested that there was a conspiracy to depopulate African Americans by manufacturers and the government. In addition, individual lifestyle choices, alcohol use, and religious factors were perceived as a cause of diabetes. There were also some fatalistic beliefs about diabetes being caused by curses or passed down from ancestors. (Table 2). This is reflected in the quote below.

**Table 2:** African Americans perception of the cause of type 2 diabetes.

Theme	Causal factor	Sample quotes
**General reasons for diabetes**	Different things altogether causes diabetes.	**DBPID_0009:…**it's a different variety of things that I hear.. that cause it, and so it ain't no one thing that I'm looking at. It's seem like to me, everything…anything
		**DBPID_001:** … it's just a lot of causes too and not just, oh, you obese.
**Biology and Genetics**	Body make-up of the individual	**DBPID_001:** I personally think that diabetes is unique to the person. So it has to do with that person's chemical makeup.
	Hormonal factors	**DBPID_0013:** It's hormonal changes. Because diabetes is, has to deal with the hormone, the endocrine system, hormone changes in the body.
	Pregnancy	**DBPID_002**: Yeah. I was at old age when I got pregnant, so I went into a different, .. body frame. Then I got diabetes when I had him (son). And so I thought because I got pregnant, that I became a diabetic.
	Old age	**DBPID_0020:** So it just something that just happen. When you get up in age, like me, I got my. at 45. I'll be 60 next year… when you started getting up in age, your body change. As they say, your hormones, whatever, change …
		**DBPID_0038**: You been here a half a century. What makes you think you ain't going to catch nothing? It seems like when you get in your late 40s or your middle 40s, everything going to messing up on you.
	Body not working to produce insulin	**DBPID_002:** I'm thinking .. there's something in your body that is not going to work right, like it won't produce insulin. Well, that's your body. That's not your lifestyle. And you're going to get it if you can't produce insulin. I don't care if you eat whatever.
	Family had it/Hereditary/Genetic	**DBPID_001:** I knew it was hereditary… one of your parents had that, then it was a good possibility that you could, get it, Type 2, at some point. So my father and… my paternal grandmother both had it.
		**DBPID_004:** So it was probably going to catch up with me, because I have two brothers and my mother are diabetic. Hereditary. It's in the genes, in the family.
	Weight gain and being overweight	**DBPID_001:** For me it would have been a weight gain. .. they were basically telling me that because my weight was out of control. And my weight was basically increasing the risk of me getting the diabetes. And so over the years, I continued to gain weight.
		**DBPID_003:** I think mine has a lot to do with the weight though. Because when I was smaller, I didn't have it. But I've gotten bigger over the last few years, and that's when I developed it.
**Medications**	Vaccines	**DBPID_0038…** it's either the food or the medicine, one or the other. High corporates, big money grabbers… you had to do what they told you to do. Regardless of what could be the repercussions… Forty years ago you took a measles shot, you took a mumps shot, you put that little cube of sugar in your mouth. You don't know if that has something to do… they might not even have them records from 50-some years ago of the medicine that we took back then?.. And what was in it? .. it could be all that medicine… at that time, drawing us to be where we are today. … Who's to say what's really in the medicine? We don't make the medicine.
		**DBPID_0041:.** this .. so-called medicine… In the vials, who's watching them…Who's these people making it? We don't know who's making it… the medicine, it was stuff over the years that they tell us we have to take this shot for this, this, this, but then when they take it even the vaccine for chickenpox, or you need a vaccine for mumps or whatever, you need vaccines for most other things… ..Well, if you got people shooting you up with stuff, that's what they determine you going to have diabetes… We didn't know nothing about it. Get in there, boy, and get that shot. it's trickery. They fooled us.
	Side effects from medications	**DBPID_0013:** the psychotropic drugs, the medications they give you for the mental health… It causes diabetes. I was on steroids, and I was taking psychotropic drugs, and .. that's when I became a borderline diabetic. Psych medication makes you eat, blow up. … I was hungry all the time. I was urinating all the time. It made me diabetic. Steroids cause it, cause diabetes.
**Psychological**	Depression	**DBPID_0013:** So when my son died, depression set in on me, and I was inactive. I was mostly laying down, ..and didn't care of what I ate. I wasn't moving around, wasn't doing what I usually did. So I'm thinking that that is when my diabetes set in there.
	Stress	**WOMAN 4:** I think mine was stress-induced…at the time that I had found out I had diabetes, my meal everyday was peppermints and Pepsi. And I was working in a daycare, and that's all I was able to pop them peppermints in my mouth all day long and drink a Pepsi, . . . .., and it was a stressful time in my life. So it was probably going to catch up with me, because I have two brothers and my mother are diabetic. Yeah. And then I think stress just helped pull that trigger.
		**DBPID_0020:** I think stress too. Stress would cause a lot of diseases that are dormant in your body.
**Environment**	Diabetes comes from the air	**DBPID_0041**: It (Diabetes) could be in the air.
		**DBPID_0038:** It (Diabetes) could be in the air too. Just like we go outside and get the flu, you know, the same thing. It can happen. It could be in the air.
**Sociocultural factors**	African Americans’ eating habits from growing up	**DBPID_0018**: That's what's in the food. You know, like they said, we were talking about like how you grew up, what you ate, what was left over, you know.
	African Americans’ culture and cultural food caused diabetes	**DBPID_0038:…**something that it was eating that cause this. Our cultural food. I tell you. Well, we, the average black person eat neck bones. The average black the person eat chitlins. The average black person eat hog bones…The average black person eat fried pork chops, baked pork chops, pork, basically. Some people can eat it and it don't bother them. … but I'm just saying that's our culture of food. But I don't eat it no more, and I should have had that much sense years ago
		**DBPID_0025:**..They say that, you know, it's more widely spread right now because of our habits, our culture, the way we come up.
	From ancestors	**DBPID_0013:** Diabetes come from our ancestors
		**DBPID_006:** I think there was some things in there, based upon, you know, the days of our ancestor that was transferred into our DNA and our genes that contribute to diabetes in these days. I truly do.
	Curses	**DBPID_006:** I just think it's a generational curse. It's in your family, and sometimes it skips people. Like it's five of us, and none of my sisters and brothers got it, but I got it. … I don't know why I was the lucky one… A generational curse, which, kind of runs through your family.
	Conspiracy to depopulate African Americans by corporates working together with the government and manufacturers to include something in food, medicine or vaccines	**DBPID_0038:** If there's no war, then they got to thin out the population. So if you put chemicals in food, that's another way to thin out the population. That's what I'm going along with, because, .. when I was a kid, if I got diabetes now, why didn't I have a trace of it when I was a kid? That's just like if you got pneumonia or chicken pox or something like that. You got a trace in you. There's got to be something that the manufacturer is putting in this food. There's got to be something in the food..that they… feeding us in the hood. Because when we was kids, …We had no problem. We could eat anything. You could eat ten pork chops and never get no high blood pressure.. There's something in the medicine. It's all about getting paid.or it could be the government… The United States .. don't want to have the cure for it, because it's another way to thin out the population. Why is it turning to the blacks? Why is it turning to the majority of us to have diabetes, high blood pressure and sugar diabetes and that and the high blood pressure and all the rest of the package?
		**DBPID_0041:** Killing off the population…Or they can target one area and put certain things in one area like this certain kind of food,.. then it spreads out like that… like he said, control the population… they put it in the babies. The baby needs a shot… for this, or you need a shot for that. Why? All these years I'm going to take these shots.
**Lifestyle choices**	Eating too much sugar	**DBPID_0024:** Sugar. Eating sugary food …
		**WOMAN:** Sugar. Yep. A lot of sugars.
	Lack of exercise	**DBPID_005:** What you eat, your exercise, your lifestyle. It ain't your lifestyle along with your friends that's going out to eat with you, because it can only affect you. They can sit there, eat cake and ice cream and big hamburgers and French fries, and be fine. But with you, your sugar is all up.. It's your individual lifestyle then.
		**DBPID_0037:** I went to the doctor one day, and he said I had diabetes. And I said how did I get it? He said from being overweight, you know, not doing enough exercise.
	Wrong diet (Fatty foods, Junk food, Candy)	**DBPID_0012:** The wrong diet. Fatty foods. Too much what the thing is, fruits.
		**DBPID_0028:** I think I was overweight and I was eating so much candy and junk food and all that stuff. And not eating right.
**Religion/Spiritual beliefs**	God gave diabetes	**DBPID_0014**: .. I blamed God. I was angry with God. But as I talked to peoples, and I talked to the pastor, I was angry, because I said, why me, God? I try to walk in your Christian way. I don't drink. I don't smoke. I don't fornicate. You know, I don't do any of those things. So, God, why me? Ask God questions… that's what God is there for. Why me, God, you know?
	Punishment from God	**DBPID_0025:** I think I'm being punished or something because no one in my family has it. I eat healthy, you know… It would have to be, probably God. Something I did that I'm not aware or not familiar with or remember, … because I'm not a bad person, never have been. So I try my best to do everything good right now, help everybody, because, again, I believe I'm being punished for some, no one in my family has this. Again, I felt, I'm going to say, that I was being punished for something that I did… I didn't cause myself to have diabetes …
**Drugs and substances**	Chemicals in the food being eaten	**DBPID_0041**:..when you don't raise your own animal, so you don't know what they shooting them animals up with. You don't know what they put on your stuff… they start injecting these animals with different hormones and different kind of stuff that's unnatural … It's got to be the food… we don't grow our own food…so we don't know what we're getting.
		**DBPID_0038:** The chemicals. Because if you really look at it, people back in the days grew their own food. They didn't have no diabetes and none of that crap. They didn't. You didn't hear them talk about no diabetes. It's in the food. It's got to be chemicals in the food. … Food or the medicine. It had to be the food, because black people have they own remedies of how they took care of they self…. .it's either the food or the medicine, one or the other… because you've got so many people on the medicine now… you've got kids these days that's diabetics…So there's something in that food, man.
	Alcohol abuse	**DBPID_0035:** Alcohol abuse… I noticed that when I was drinking…I'd lost weight, but I still had diabetes. It was because of the sugar intake of the liquor.
		**DBPID_0024:** I drink beer. Look, I'm going to tell you…. I could've had diabetes from drinking, because when I was five years old, when I was six months … I came here from Tennessee, and my daddy had a '64 Chevy Impala, and he had no friends. So when I turned five, he used to beat me to make me drink, so I was drinking Pabst Blue Ribbon at the age of five…and I've been drinking ever since.

*DBPID_0017: I got diabetes through medication. The name of the medication is Risperdal. Years ago, they gave us Risperdal. Now they didn't tell me the side effects of it. Because I should not get this disease… I think a lot of diabetes in our black communities is due to medication … I promise you got it through meds*.

While some participants expressed beliefs that diabetes was caused by God’s punishment for their sins, some participants disagreed. Participants stated:

*DBPID_0017: …God didn't give me this. God didn't make that medication,… no, I think God is a healer. And things happen for a reason. It's a test*.

*DBPID_0011: I have a blameless God. I can't blame my God for anything that's human created. You see, because humans make medicine, certain types of medicine… So my God is blameless… I would never blame my God, because God has nothing to do with human, I mean, we were born imperfect, and we will die, and we will falter*, *and we will get sick*

#### CSM domain: Perception of treatment and personal control of diabetes

In general, participants were unsure of how to manage and control diabetes. However, in terms of treatment control, some participants believed medications could help control diabetes. For example, there were statements such as,

*DBPID_003*: *The pills can help you maintain diabetes…*

*DBPID_0017: …once I got back on metformin, I started taking control*.

Participants described various coping behaviors that enhanced their personal control of diabetes including internal control through empowerment and positive attitudes, and external control through faith in God and support from peers.

*DBPID_0011: …my grandmother told me, if you do not self-educate yourself*, *I feel sorry for you. And she told me..if you let inequality of the races stop you from being who you are as a strong, black woman, she said, more better for you… she told me you cannot let that stop you from being you and taking care of yourself …*

In terms of internal control, coping mechanisms included self-education, empowerment of oneself, and ‘taking care of oneself’ through self-monitoring and self-regulation. This is reflected in the quote below.

*DBPID_001: …if you can train yourself to know the signs of what activates, what elevates your diabetes, and what does not, I think you can live a normal life if you learn that*.

In addition, internal control related to a need to focus on positive attitudes and not the negativity associated with diabetes. Maintaining a positive outlook regarding living and coping with diabetes was important to AAs because it allowed them to feel less defeated and in control and able to prevent diabetes complications. The two statements below illustrated this.

*DBPID_0011: I can't think negative about it… So what I do is I try to find ways of survival… in my own mind, if I focus on the negative that it does to me, then I feel like I'm defeated, that I'm allowing the disease to take control of me… In this life, it's very hard, let alone having diabetes, and let alone being black and female in this country. So I can't afford to have something working against me, so I try to find the positives, and I try to do what I can … I've had to accept the things that I can change, and I have to have the courage to go through the things that I can't. I'm going to be all right. I ain't going to worry about it*. *…*

*DBPID_0038: I tell my girlfriend, I said, listen, babe, I'm not going to argue with you. …t's better for me… if I sit here and argue with you, I'm going to have a stroke, I ain't going to let nothing worry me… You can have a heart attack … I'm worrying about things that I can't change, and it making me sick … So I said, no, I'm not going to let nothing worry me. I don't care*.

In terms of external control, participants reported that their faith took away worries about diabetes, provided knowledge and confidence in managing diabetes, and shifted control of the disease to God.

*DBPID_0015: Well, I believe, for me, that God plays a tremendous part in all of this. He gives me more knowledge. He takes me through this diabetes. God has a lot to do with it, but he carries me through it*.

*DBPID_0038: If you got faith in God, yeah, that helps. It ease your mind … if you believe in God, you got faith in God… . I would say, okay, good Lord, I have no control over this. All I can do is take my medicine. I'm putting this in your hands … So if I got faith in you, and I'm putting it in your hand, I'm not going to worry as much, because I know you got control of it. I got faith in you. You going to take care of me*.

Additionally, participants believed external support from the AA community through discussion groups and networking with faith-believing peers helped control diabetes. Participants stated:

*DBPID_0011: …It is so hard to have a whole bunch of black people together to strengthen one another… these type of groups that you're having today, is necessary for the survival of people with diabetes, because networking and being together as a group is more powerful than trying to deal with it as one…*.

*DBPID_0018: I'm Baha'i… when you have a support network outside of your family and your doctor, and you have support from your faith, people in your faith, that really helps*.

#### CSM domain: Perceived consequences of diabetes

Participants’ reported that they experienced relationship, lifestyle, and sociocultural consequences, due to having diabetes.

*Relationship consequences* included changes in sexual and family/friends relations. For example, having diabetes made participants’ feel infantilized and controlled which affected their sexual relationships and changed their identity as a sexual person. Participants reported an inability to find a significant other or enjoy family times/social gatherings. In addition, participants reported a shift in internal locus of control (their ability to influence the outcomes of the disease) to external locus of control (blame outside forces). Because of the shift in perceived control, participants tended to demonize the disease. These themes are reflected in the quotes below:

Sexual relationships

*DBPID_006: You don't be feeling it all the time like you used to… With my family and my woman. You know, like they think you're a diabetic because you don't have no control over your life. And they like dictate you … you be in the restaurant ordering. They say, you can't have that… it changed the other person. They don't feel the same about me no more. They ain't paying attention to me no more*.

*DBPID_0018: Well, for me, like he said, like the sexual drive, you know, it was a thing where just, you know, don't touch me*.

Friends/family relationships

*DBPID_004: Or …you go out to eat with the girls, and they like… you shouldn't be drinking soda. You should be drinking water… you shouldn't even be thinking about dessert, or drinking…You know, yeah, I know … Thank you for being concerned, but I have this*.

*DBPID_006: And diabetes changes your family, because once they know you got it, they like always on me. Like if we go to a family event, I can't have anything. I can’t even enjoy myself. …And like my young daughter, she going to have a baby… And we was talking, and my sugar got low. I said, I know what to do when my sugar gets low … Then she went on to, oh, when my baby get here I don't know if I'm going to let you watch him. Because… You might fall out or stuff. I say, girl, I had you, so I didn't fall out when we were raising you, right? …*.

On the contrary, a couple of participants disagreed that having diabetes affected their relationships. Participants stated:

*DBPID_0025: I beg to differ on that, because my fiancé wants to take care of me. He lives in (town name), and he's begging me to move down there every day*.

*DBPID_0034: I disagree with him. And the reason I'm disagreeing, because I … I got plenty of friends, and I got female friends that I deal with… I don't let my illness come between that*.

*Lifestyle changes* included how diabetes affected their whole life (eating habits, exercise), and changed how they enjoyed/loved food. Having diabetes also changed their lifestyle by affecting their job and functional roles, influencing the type of job that they could have, hence changing their identity as an employee and a social person. Sample quotes included:

Functional roles

*DBPID_0017: I used to be a chef, so I can't do what I love to do to since four years old is cook, so it's life-changing for me and my family. I don't volunteer in the community no more*. *It just you have to say no to a lot of things… I was can I say mad as hell?*

*DBPID_0018: …I don't have the attention span to stay awake a long time after being at work all day. I just want to go home … Yeah. I'm very much, you know, social but a loner … I just don't have the mental capacity to do all that anymore*.

Experiences with Food

*DBPID_003: I used to enjoy food, and I don't have that luxury anymore. That's gone*.

*DBPID_006: Well, like food is your enemy now*.

Change of identity as an employee

*DBPID_006: Well, diabetes, it messed up my job, because I used to be a truck driver, and once I got diabetes, they'd want to take me off the road because I didn't have control over it… so I had to change jobs because of diabetes*.

*Sociocultural consequences* included diabetes made the African American family bonding experience of eating difficult, and the disease took away from their cultural experiences.

*DBPID_0009: So I'm finding it very difficult to stay away from those foods, because you have your loved ones … they sitting there eating it, and you can't have it. That is very, very difficult… what I still find difficult for myself today is… that you can't eat certain foods forever. Now you know as a black, African-American, we like the type of foods that we grew up with*. *So I'm finding it very difficult to stay away from those foods…*

*DBPID_003: The food that you like, so the things that you were brought up on. So all of your cultural things are out the window*. *You know, they're like the forbidden…*

Finally, participants’ reported medical consequences such as diabetes complications and loss of organ function because of diabetes.

*DBPID_006: And diabetes make you have other problems… My eyes got real bad. And I had… Acid reflux, that was real bad. I couldn't even drink milk, and serious stuff would just burn in your chest… I feel like I’m having a heart attack or something*..

Other perceptions of diabetes related to emotional representations about having diabetes and concerns about the disease. For example, participants were asked ‘when you think of the word diabetes what one word describes it’. Participants mostly used a variety of words related to their emotional representation of the disease. (Table 3).

**Table 3:** African Americans one word used to describe type 2 diabetes.

Illness Perception Domain	Descriptions
**Cause of diabetes**	Hereditary
**Emotional representation of diabetes**	Physically challenging
	Complicated
	Life-changing
	Acceptance
	Horrible
	Dangerous
	Death/Deadly
	Frightening
	Very serious
	Painful
	Hard
	Scary
	Challenging
	Struggle
	Unpredictable
	Tiring
	Terrible
	Worrisome
	Sour
	Controlling
	Frustrating
	Made food your enemy
**Identity/Symptoms of diabetes**	Irregular weight
	Pain

### Emotional representations of diabetes

Participants reported a variety of fear-based reactions to having diabetes, including a fear of diabetes diagnosis among family members, fear and doubt of their future, and fear of death, amputation and blindness due to diabetes complications. There were also expressions of anger and frustration because it was hard to accept the diabetes diagnosis, the lifestyle changes, and the need to control diabetes. Participants also reported experiencing emotional dysregulation and depression. These themes are reflected in the quotes below:

#### Fear of diabetes diagnosis and diabetes complications

*DBPID_006: My daughter expect to get it now. You know, she's pregnant now, but she always asking me about my diabetes… she's so scared of getting it … I think diabetes… scare you at first … like if you see somebody with their limbs off, it's usually diabetes, they ain't got no foot. It was usually diabetes that did that… or I can't see now, I'm blind, because I had diabetes. And then you'll be more, but I don't want to be like that*.

*MAN 2: …So my brother, my sister, my daddy, they all have diabetes too. Right now, my father… to look at his legs… these people are talking about cutting… My brother, now they cutting on his feet… So I'm kind of wondering… I'm going to be in that situation one day… looking at my dad right now, getting ready to look like he's going to lose his legs. And then my brother already lost three toes… it scared the shit out of me… it scared me to death*.

#### Fear and doubt of the future

*DBPID_0038: Me, I can't see what the future going to bring. All I can do is live right now, try to take care of this and make it to the future. Once I make it there, then I can keep going on, simple as that… I can't see it. I take it one day at a time*.

*DBPID_0041: I want to be happy and then, my future … I want to be able to see my grandkids get older and graduate and stuff like that. That is my major … as long as I can talk to them and go see them and they going to see their papa*.

#### Anger and frustration with diabetes

*DBPID_0020: When I got it, it was hard for me to accept it. I was angry… because I couldn't face the fact how come I have to be a diabetic, and I got to change all the things that I'm used to doing and should rearrange my whole life? And I was upset. It was hard for me*.

*MAN 2: I'm about ready to give up… I am so sick and tired of… my sugar up that high … I don't eat too much sugar or eat no candy. I don't eat no sodas and stuff … and my sugar is steady going up on me*. *I'm trying to do what they tell me to do… but I can't do it…*

Emotional dysregulation and depression

*DBPID_003: Sometimes your mood go up and down. Sometimes you have a good day, you know, nothing bother you, like sometimes you might have pain or burning in your feet. Some days you might not have that, you know. Seems like you be more angry*.

*DBPID_0025: My doctor tells me, you know, [DBPID_0025], you're kind of going into depression a little bit, because I stay in the bed all the time, because the neuropathy hurts so bad*.

On the other hand, some participants expressed how diabetes had not influenced them negatively. For example, some AAs reported that diabetes did not control their emotions or make them depressed/upset. These individuals were not worried about the disease, but had learned to be positive, control diabetes, and live life despite the disease.

*DBPID_002*: *I'm not going to let diabetes control me*. *My mom had diabetes*. *My dad had diabetes*. *My brother had diabetes*. *But I refuse to get myself depressed and upset because I have diabetes… I'm moving along with my life… I Live and let live*. *I'm not going to worry about it* … *you let the Lord handle it*. *He going to call you home when it's your time*.

*DBPID_0020*: *…I might have diabetes*, *but diabetes don't have me*. *I live my life and do what I got to do*. *Don't let it get you down*, *because it get you down*, *you'll be depressed*. *Keep doing what you doing*. *Eat right*, *exercise*, *and live a long life*. *Some people live to be 100 with diabetes*. *Some live longer than that*. *So I learned to say I got diabetes… but it don't have me*. *I'm not going to claim it*, *because I'm going to keep on living*.

#### Concerns about diabetes

Participants’ reported being worried about their family members and themselves dying from diabetes. Diabetes was perceived as a distal cause of death. Observing the death of family members diagnosed with diabetes reinforced participants’ belief about the severity of the disease. Concern about dying also motivated AAs to want to prevent the disease in their children and grandchildren. A participant stated:

*DBPID_0016: …a few of my family members have died from it (diabetes) … I was talking to one of my brothers at night … The next morning, he was gone. My sister had a stroke, and she stayed in a coma for about maybe about a year, and she didn't die until they moved her to (town name)… a couple days later after that, she passed away … So I'm still kind of angry, because I feel like if somebody could reach down and tell me this (diabetes information), maybe I could get a better handle on my disease and be able to explain it better to my grandchildren or whatever*.

## Discussion

To our knowledge, this is the first study to explore the underlying beliefs of AAs with type 2 diabetes using the CSM theoretical framework. The study findings showed that AAs illness perceptions are complex and intertwined with sociocultural and psychosocial issues, which may not easily fit into the biomedical perception of diabetes. Using the CSM helped illuminate AA beliefs about diabetes based on their perceptions of the symptoms experienced, the timeline of diabetes, cause(s) of diabetes, treatment and personal control of diabetes, and the consequences of diabetes. Participants also reported experiencing emotional distress, fear, and concerns about diabetes complications and death, as well as anger and frustration due to having diabetes.

### Symptoms and timeline of diabetes

Participants identified a variety of symptoms that they experienced before and after the diabetes diagnosis, and believed that diabetes would last for a while. On the other hand, some participants believed that diabetes would go away with exercise and weight loss. How patients interpret changes in their bodies and whether these changes signal diabetes as a long-term serious chronic disease have implications for how and what medications patients take to maintain control of diabetes.[25] Similar to a prior study, [25] participants in our study experienced symptoms associated with undiagnosed diabetes (e.g., excessive thirst, frequent urination, fatigue) before the diabetes diagnosis. It is unclear if the AA patients in this study had symptoms of diabetes that they ignored, or if they simply did not recognize as related to diabetes. Skelly et al., 2006 noted that AAs’ knowledge of the symptoms of diabetes during diabetes onset might be interchanged with the symptoms of complications. In a study of AA rural adults, knowledge of symptoms was learned from what had been observed in family and friends. [8] Educating AAs about the symptoms of diabetes, especially, if these patients are borderline diabetic is paramount to patient realization of diabetes as a chronic disease that is long-term and that requires treatment control and self-management.

### Cause of diabetes

There were treatment and etiology misperceptions among AAs in this study possibly due to a lack of knowledge and confusion regarding the cause of diabetes. Participants perceived that medications prescribed for controlling diabetes, including its side effects, caused diabetes. Participants also articulated that diabetes occurred because of curses and ancestors. In our prior work, AAs with diabetes reported a sociocultural component to their perception of the development of diabetes, with an attribution to stigma among family members and the community, that led to a denial of the disease.[49]

Similar to our current study, Egede et al., 2003 showed that AAs perceive diabetes as a curse passed on from generation to generation. [50] In our current study, some participants believed God influenced how they developed diabetes and that the government and health providers hiding diabetes management information from them led to the disease. Participants also suggested that diabetes was a conspiracy by manufacturers and the government to depopulate AAs. A prior study has documented that AAs sometimes perceive the federal government as using chronic diseases to kill and wipe out black populations. This mistrust stems from the historical and current racial discriminations faced by AAs with the US healthcare system. [51, 52]

The current study’s finding of misperceptions are consistent with a prior study that showed how dimensions of fatalism are embedded in patients’ meaning of diabetes, their illness experience, their coping responses/behavior, and their spiritual beliefs. [50] Fatalism, defined as “a complex psychological cycle characterized by perceptions of hopelessness, worthlessness, meaningless, powerlessness, and social despair”[53] was evident in AAs’ perception of the cause of diabetes. For providers, exploring with patients why they believe they developed diabetes may highlight important disconnects between the biomedical understanding of disease etiology and patient views of the cause of their diabetes. Providers need to be mindful of how they are educating AA patients with diabetes on the cause of their disease. Explaining disease etiology in a simple, plain, and patient-centered manner while acknowledging that patients might have less ability to process and understand health information (low health literacy) is important. Patients are less likely to follow their treatment recommendations if they are confused by the clinical explanation of how diabetes affects their body or their provider’s explanation of what might have led to their diabetes.[25]

### Treatment and personal control of diabetes

Many AAs in this study perceived there were positive internal and external control mechanisms that helped manage diabetes including maintaining a positive outlook regarding living with diabetes, as well as spirituality and reliance on religion. Participants reported that their faith shifted the control of the disease to God. In a previous study of AAs with type 2 diabetes, participants believed that surviving with diabetes depended on God rather than their own individual efforts, and that the disease was controllable. [50] Researchers have shown that spirituality is important in how AAs cope with diabetes. [54] Samuel-Hodge et al., 2000 noted a tendency for AA women to ask for God’s help in controlling diabetes and complications. [55] Similarly, Anderson et al., 2000 suggested that a strong belief in God, along with support from church members were important to AA men and women with diabetes.[56] This current study showed that although there is a strong spiritual component to AAs’ perception of the control of diabetes; participants do not absolve themselves from personal responsibility, but believe in individual efforts such as positive thinking, self-empowerment, and self-monitoring to complement God’s work in controlling diabetes.

In addition, similar to a prior study, [8] some participants reported the use of medication to “control” or “maintain” diabetes, further showing AAs’ belief in self-management of diabetes. Patient illness perceptions are culturally constructed with meaning and have implications for the successful self-management of diabetes including medication adherence. As providers gain a deeper understanding of patient illness and treatment perceptions as a way of improving communication regarding medication use, significant attention also needs to be paid to the social, spiritual, and psychological coping mechanisms AAs use for dealing with diabetes and leveraging these mechanisms in designing culturally–appropriate interventions for AAs.[16, 25]

### Consequences of diabetes

Based on our study results, there are social and interpersonal consequences of having diabetes for AAs. Participants experienced a sense of surveillance/loss of autonomy and lifestyle changes because of having diabetes. Prior research show AAs with diabetes feel powerless over their life and future. [57] Study results show that participants perceived that diabetes changed their life by influencing how they ate favorite foods, their job and functional roles, and by changing their identity as an employee, a cultural individual, a sexual person, and a social person. In a study of the illness narratives of AA men with type 2 diabetes, Liburd et al., 2004 reported that diabetes affected employability and job security, sex appeal, and other social relationships among participants. AA men reported changing their lifestyle in order to manage diabetes. Diabetes complications impacted their ability to meet routine obligations, affecting their spouses and others, as well as themselves. In the exploration and management of AA illness perceptions, it is important to consider how diabetes significantly affects several contextual factors external to the individual, which then influences their experience of living with the disease.[25, 58] Interventions that address AAs’ diabetes management need to account for how the disease changes the individual and their interpersonal lives, and the psychological impact of these changes.

In this study, AAs reported how diabetes influenced their sociocultural context, as well as affected their need for social support. Diabetes made the family bonding experience of eating difficult, limited participants’ ability to eat their cultural foods, and diminished their cultural experiences. Prior studies have shown that AAs with diabetes have concerns such as managing their disease in a way that includes ethnic foods and participating in social gatherings involving food. [57, 59, 60]. The type of food, the consumption of food, and its role in social events are culturally important and have meaning among AAs that needs to be recognized in the development and design of culturally competent, disease management interventions and treatment plans.[57, 61] For example, enhancing positive illness perceptions can focus on changing food preparation, while still embracing AAs’ cultural experience of eating as a family. In terms of social support, it was not surprising that diabetes affected AAs relationships with family and friends as participants felt infantilized and controlled. Family support is important to AAs with diabetes, especially in relation to medication self-management. [58, 59] A sense of no support and family conflict may create a negative emotional context that discourages patients and increases the feeling of diabetes as being burdensome or intrusive. [62] Successfully managing diabetes can increase patient’s personal sense of control and emotional well-being, especially when the families of AAs with diabetes believe optimistically that a patient’s life still has meaning despite the disease. [63] It is important to design diabetes self-management interventions that enable family members and friends to be involved in positive ways and allow for the incorporation of cultural experiences that would otherwise have made diabetes management burdensome. [63]

### Emotional representation and concern about diabetes

Participants reported negative reactions to having diabetes including anger, fear, and frustration. In prior studies, AAs with diabetes report experiencing loss of normalcy and suffering, and perceived that their lifestyle changes were stressful and intrusive. [25, 50] Similarly, fear of death and complications have been reported in prior research examining the impact of diabetes among AAs.[57] In our prior work, AAs’ reasons for intentionally not taking their medicines were associated with their concerns about medication side effects, as well as fear and frustration associated with taking their diabetes medicines. [12] Managing diabetes is stressful and AAs are fearful and preoccupied with thoughts of future disability, loss of independence, and death. [25, 58] Providing behavioral and psychological support that alleviates the distress and emotional toll of diabetes, and offers positive support through family, community and social organizations are needed for AAs with type 2 diabetes.

Our study showed that participants were concerned about family members and themselves dying from diabetes, because they had observed diabetes complications and mortality in their family. Similarly, in a prior study, participants reported seeing family members and friends experience diabetes complications or die from the disease. AAs’ personal experience with diabetes is related to the experience of diabetes in their friends and family members. [50] Given the high prevalence of diabetes in the AA community and the salience of its detrimental consequences in patients’ lives, attention to the family context of disease is warranted.[25]

### Implications for practice

Healthcare providers and diabetes educators can support AAs with type 2 diabetes by acknowledging that they are aware of and sensitive to the multiple and complex psychosocial and cultural struggles that AAs must face and negotiate during disease and medication management. As shown in our study findings, for AAs with diabetes, there are several issues to consider.

To help patients implement adherence behavior changes, health care educators and providers need to be aware of how different cultural contexts influence what patients know and believe about diabetes. [8] Studies have shown that illness perceptions influence whether patients take their medicines or not, and should be taken into account in designing medication use interventions.[10, 11] Diabetes educators and providers can encourage patients to discuss their perceptions of diabetes and how having the disease influences their individuality, independence, and social and cultural contexts. In addition, providers need to pay attention to making referrals to needed specialty, community, and social services that can provide psychosocial support and enhance AAs’ positive coping skills. Peer educator, family, and/or faith support-based interventions are useful to AAs with diabetes, providing a forum for these individuals to share diabetes management struggles and strategies and provide support beyond the clinical encounter. [25]

Patient-provider behavioral interventions that negotiate and compromise on patient illness perceptions may lead to positive health behaviors including improved diabetes self-management and adherence. [64, 65]In a prior work, AAs with diabetes perceived that there was a race-mediated effect of how they developed diabetes due to poverty associated with past slavery and racial discrimination they had experienced from their health care providers. [49] Since illness perceptions are influenced by patient environmental and sociocultural contexts,[14] it follows that cultural values need to be integrated into patient-centered interventions designed to present positive illness perceptions that can enhance self-management and medication adherence. [65] Gaps in patient and provider concordance in illness perceptions exist and hinder medication adherence. [64] Hence, enhanced patient and provider communication interventions that addresses the behavioral, educational and sociocultural environment of AAs with diabetes can lead to improved adherence.

### Limitations

This study had several limitations. First, due to the high number of patients taking oral diabetes medicines compared to insulin, only patients taking oral diabetes medicines are included. Future studies will consider the inclusion of all individuals including those using insulin. Second, illness perceptions might differ between men and women and across age groups. The focus groups included a mix of men and women, but were specific to middle-aged adults. Future studies will consider possible gender differences in perceptions and include a wider age group. Finally, the authors conducted the study in a Midwestern location. It is possible that geographic regions influence beliefs, such that beliefs among African Americans in South Carolina might be different from African Americans in Chicago.

## Conclusions

According to Leventhal’s common sense model, patient illness perceptions are shaped in the sociocultural context of an individual. For African Americans with type 2 diabetes, improved diabetes self-management behaviors such as medication adherence might be influenced by the meaning the individual attaches to diabetes, the available psychosocial support for managing the disease, and patient experience with the disease.
